# Is involution effective? Research on the influence mechanism of perceived overwork climate on employees' creative performance

**DOI:** 10.3389/fpsyg.2026.1798307

**Published:** 2026-05-22

**Authors:** Yun Chen, Mengyao Zhou, Pengcheng Du, Kuntai Song, Yifei Sun

**Affiliations:** 1 School of Business, Anhui University, Anhui, China; 2 School of Management, Sun Yat-sen University, Guangzhou, China

**Keywords:** creative performance, overwork climate, prevention focus, promotion focus, trait mindfulness, work-specific

## Abstract

**Introduction:**

Amid intensifying market competition, overwork climate has become pervasive in organizations, yet its impact on employees' creative performance remains theoretically controversial. To address this gap, this study integrates Cognitive Appraisal Theory and Regulatory Focus Theory to construct a moderated mediation model, exploring how overwork climate influences employees' creative performance through work-specific regulatory focus (promotion focus vs. prevention focus) and the moderating role of trait mindfulness.

**Design/methodology/approach:**

A two-wave questionnaire survey was conducted with 597 R&D employees from Chinese technology firms, and data were analyzed using hierarchical regression and bootstrap methods.

**Findings:**

Overwork climate has a differential impact on employees' creative performance through work-specific regulatory focus, employees' trait mindfulness moderates this process. Specifically, high trait mindfulness strengthens the positive mediating role of promotion focus, whereas low trait mindfulness amplifies the negative mediating role of prevention focus.

**Discussion:**

This research elucidates the intricate mechanism and boundary conditions of the influence of the overwork climate on employees' creative performance. It resolves the conflicting findings regarding the relationship between the overwork climate and creative performance to a certain degree, uncovers the dual effects of such a climate, enriches the research in this field within an Eastern context, and facilitates the integration of the Cognitive Appraisal Theory and Regulatory Focus Theory.

## 1 Introduction

In an era marked by volatile external environments and unpredictable global economic trends, organizations face unprecedented competitive pressures. Sustaining competitive advantages and navigating crises hinges on continuous innovation (Jung et al., 2025). Stimulating employees' innovative potential and enhancing their creative performance have thus become a strategic imperative for organizational survival and development (Madjar et al., 2002; Liu et al., 2025). Since creative performance improvement typically requires sufficient time investment (Amabile and Pratt, 2016), many organizations have adopted intensified work engagement to pursue breakthrough innovations, this trend has directly fostered the formation and normalization of an overwork climate (Song et al., 2026). A prominent example is the prevalence of the “996” work schedule (9 AM to 9 PM, 6 days a week) among Chinese enterprises, which has transformed overwork from isolated individual behaviors into a pervasive organizational climate (Chen et al., 2023).

Against this background, a core question remains unclear: Is involution-style overwork climate effective for promoting employees' creative performance? As a critical situational factor, organizational climate profoundly affects employees' psychological processes and behavioral outputs (Güven and Dogan, 2025). However, existing research lacks in-depth exploration of the relationship between overwork climate and employees' creative performance, with relevant studies yielding contradictory conclusions. On the one hand, some studies have found that overwork climate leads to occupational burnout (Lazauskaite-Zabielske et al., 2023), work-home conflict (Žiedelis et al., 2024), and reduced job satisfaction (Chen et al., 2023) among employees. These adverse effects deplete the cognitive resources essential for innovation, ultimately suppressing creative performance (Lim, 2018). On the other hand, a minority of studies propose a counterview: for example, Song et al. (2026) drawing on Resource Conservation Theory, found through empirical research that an overwork climate can accumulate innovation resources through employees' proactive procrastination, thereby enhancing creativity. Zeng et al. (2025) empirical study also indicates that an overwork climate enhances the innovation performance of researchers.

The primary reasons for this controversy may lie in the following: First, existing research has primarily examined the effects of overwork climate through single-perspective frameworks such as the Situational Intensity Theory (Lazauskaite-Zabielske et al., 2023) and Resource Conservation Theory (Song et al., 2026) focusing solely on single mediating pathways such as psychological detachment and proactive procrastination, while overlooking the potential multiple mediating mechanisms between the two. Second, existing research has paid insufficient attention to individual differences in the relationship between the two. Although some studies have examined the interaction between an overwork climate and traits such as perfectionism (Mazzetti et al., 2014), they have focused on other outcome variables and failed to reveal why the same overwork climate leads to heterogeneous creative performance. Finally, existing research samples are largely confined to Western workplaces (Lazauskaite-Zabielske et al., 2023; Žiedelis et al., 2024) or general positions in Chinese workplaces (Song et al., 2026), with targeted studies on specific industries or job groups being scarce. While prior research has found that regional cultural factors (Takagi et al., 2025) and occupational characteristics (Zeng et al., 2025) influence the effects of an overwork climate, current studies lack contextualized exploration of specific industries in China. These limitations collectively contribute to significant academic controversy regarding the relationship between overwork climate and employees' creative performance. There is an urgent need to integrate multiple theoretical perspectives and systematically examine the combined effects of individual traits and multiple mediating mechanisms to more comprehensively reveal the boundary conditions and underlying logic of this relationship.

The Cognitive Appraisal Theory posits that individuals with different personality traits will perceive and interpret the same stressor in distinct ways, leading to variations in its impact (Podsakoff N et al., 2023). In organizations with strong overwork climate, employees' attention is often consumed by heavy workloads, which can easily exacerbate workaholism and emotional exhaustion (Paškvan et al., 2016). As trait mindfulness that can alleviate stress experiences through attentional monitoring and non-judgmental acceptance (Puhlmann L M and Engert, 2025), trait mindfulness may help employees temporarily detach from excessive preoccupation with their workload, observe their emotional reactions with a non-judgmental attitude, and rationally assess the overwork climate. In other words, as a variable of individual difference, trait mindfulness may moderate the relationship between an overwork climate and creative performance by influencing employees' cognitive evaluations of that climate.

So, when faced with the specific stressor of overwork climate, how do employees with different levels of trait mindfulness assess and interpret it? The Cognitive Appraisal Theory offers an important analytical perspective on this issue. This theory posits that when individuals encounter stressful situations, they first engage in cognitive evaluation, which can generally be categorized into two main types: challenge-oriented and obstacle-oriented cognitive evaluations (Paškvan et al., 2016). However, within this theoretical framework, the specific factors and conditions that lead individuals to form challenge-oriented or obstacle-oriented cognitions, as well as the underlying evaluation criteria and formation mechanisms, have not yet been fully clarified or refined. The Regulatory Focus Theory can supplement this. This theory suggests that an individual's assessment of the gains and losses associated with a stressor in a stressful situation plays a crucial role in shaping their stress regulation strategies. When individuals perceive potential benefits from a stressor, they adopt promotion focus; conversely, if they anticipate losses, they adopt prevention focus. These two distinct regulatory focuses lead to differences in creative performance (Higgins, 1997). In the workplace, employees' regulatory focus is influenced by a combination of individual traits and the work environment. In other words, work-specific regulatory focus is a context-sensitive but moderately stable self-regulatory orientation (Wallace and Chen, 2006). Based on this, this study posits that the interaction between an overwork climate and trait mindfulness may lead employees to evaluate the earnings and losses of this stressor, resulting in different work-specific regulatory focus, thereby resulting in varying levels of creative performance.

In summary, this study draws on Cognitive Appraisal Theory and Regulatory Focus Theory to systematically explore how employees' perceived overwork climate influences their creative performance. Given the pervasive climate of overwork in China's high-tech industry (Li et al., 2018) and the sector's heightened demand for innovation, this study selected R&D personnel in China's high-tech industry as its research sample. By integrating trait factors with stressors self-regulatory strategies, the study clearly addresses the core question of “when does overwork climate promote and when does it inhibit creative performance.” This study resolves the conflicting conclusions regarding the relationship between overwork climate and creative performance, reveals the dual effects of such a climate, enriches Eastern-contextualized research in this field, and promotes the integration of Cognitive Appraisal Theory and Regulatory Focus Theory.

## 2 Theoretical foundations and research hypotheses

### 2.1 Theoretical foundations

An overwork climate refers to the shared cognitive perception employees form regarding overwork-related practices and expectations within the work climate. Its core logic lies in the fact that organizations, through policies, managerial behaviors, and cultural signals, shape inputs exceeding routine work requirements—such as overtime, taking work home, and working on weekends—into necessary conditions for achieving success, promotion, or recognition. This culture is not merely a single requirement for overtime work, but rather a mutually reinforcing perceptual system that encompasses expectations, encouragement, recognition, and implicit pressure regarding overwork (Mazzetti et al., 2014, 2016). To analyze its complex impact on creative performance, this study constructs an integrated theoretical framework by combining Cognitive Appraisal Theory (CAT) and Regulation Focus Theory (RFT).

Cognitive Appraisal Theory posits that stressors themselves have no fixed attributes, their impact on individuals depends on the individual's cognitive appraisal of the stressor, which is moderated by individual traits (Podsakoff N et al., 2023). The Regulation Focus Theory posits that an individual's motivational system comprises two orientations: a promotion focus, centered on “pursuing gains and achieving ideal goals,” and a prevention focus, centered on “avoiding losses and maintaining a state of safety.” The activation of these two focuses is essentially the result of an individual's cognitive trade-off between “potential gains” and “potential losses.” In other words, if an individual assesses that the climate will bring benefits and help achieve ideals, a promotion focus will form; conversely, if the individual assesses that the climate will result in losses and threaten safety, a prevention focus will form (Wallace and Chen, 2006).

Based on this, this study posits that employees' cognitive evaluation of an overwork climate is essentially a process of weighing the “potential gains” and “potential losses” inherent in overwork climate. Trait mindfulness activates different situational regulatory focuses by moderating the bias in this “gain-loss” trade-off. Under different situational regulatory focuses, employees will exhibit varying levels of creative performance.

### 2.2 Research hypotheses

#### 2.2.1 The interactive effect of overwork climate and trait mindfulness on work-specific regulatory focus

An overwork climate, as a significant source of stress, has the potential to be evaluated in two ways: it can be perceived either as a challenge or as a threat (Zeng et al., 2025). According to Cognitive Appraisal Theory, this evaluation is not an inherent property of the climate but is shaped by individual differences (Podsakoff N et al., 2023). Trait mindfulness plays a key role in this appraisal process, influencing employees' “gain-loss” assessment of the work demands associated with an overwork climate.

Employees with high levels of trait mindfulness demonstrate superior cognitive regulation and emotional balance (Brown and Ryan, 2003). When faced with a work climate characterized by an overwork climate, employees with high trait mindfulness are more inclined to assess this overall climate with caution and objectivity, rather than being swept up by it and generating instinctive stress responses (Shapiro S et al., 2006). They do not fall into anxiety or resistance due to the perception of a pervasive high-intensity work climate within the organization; instead, they are able to keenly identify potential benefits within this overall climate. Specifically, in an overwork climate, employees often need to exert extra effort at work, and these efforts are closely linked to potential benefits such as promotions, pay raises, skill enhancement, and the achievement of career goals (Zeng et al., 2025). According to Regulation Focus Theory (RFT), if an individual assesses that the environment will bring them benefits and help them achieve their ideals, they will develop a promotion focus (Higgins, 1998).

Conversely, employees with lower levels of trait mindfulness tend toward automatic, reactive thinking patterns (Lal and Jayan, 2019), which prevents them from rationally viewing the overall characteristics of an overwork climate. Furthermore, due to a lack of sufficient cognitive resources to effectively cope with stressors (Tang and Braver, 2020), they are more prone to magnifying the potential losses inherent in an overwork climate—such as physical and mental health issues (Westover, 2024), work-family conflict (Žiedelis et al., 2024), and low work engagement (Mazzetti et al., 2020) caused by excessive work. According to Regulation Focus Theory, if employees assess that the climate will result in losses or threaten their safety, they will develop a prevention focus (Wallace et al., 2009).

In summary, trait mindfulness moderates the relationship between overwork climate and work-specific regulatory focus by determining the gains and losses associated with cognitive evaluations. These evaluations, in turn, determine whether a promotion focus or a prevention focus is activated. Therefore, the impact of an overwork climate on work-specific regulatory focus is not uniform but depends on employees' levels of trait mindfulness. Based on this reasoning, we propose the following hypotheses:

H1a: For employees with high levels of trait mindfulness, an overwork climate is positively associated with activation of a promotion focus.

H1b: For employees with low levels of trait mindfulness, an overwork climate is positively associated with activation of a prevention focus.

#### 2.2.2 The relationship between work-specific regulatory focus and employees' creative performance

Guided by Regulatory Focus Theory, individuals with different focus states show significant differences in motivation and attitudes toward change (Qian et al., 2024), which may influence their creative performance.

On the one hand, when employees are in a promotion focus state, they are typically driven by self-development and achievement motivation (Higgins, 1997). Strong internal motivation is a crucial condition for generating high creative performance (Amabile, 1983) Conversely, when employees are in a prevention focus state, they often pursue “ought self”—a self-concept based on norms and expectations, which is usually associated with fulfilling duties, obligations, and responsibilities (Li et al., 2018). In this state, employees' intrinsic motivation levels are low, weakening their engagement and commitment to innovation activities, resulting in relatively lower creative performance (Idrovo et al., 2025). On the one hand, individuals in a promotion focus state prioritize pursuing benefits over worrying about failures, showing lower sensitivity to risks and often demonstrating higher willingness to change (Brockner et al., 2004). Innovation activities are often accompanied by risks, and the willingness of individuals to accept change under the state of regulation focus may lead to higher creative performance (Lanaj et al., 2012). In contrast, employees with a prevention focus usually show lower risk preference and adopt preventive strategies to significantly reduce potential losses and failure probabilities (Qian et al., 2024). During problem-solving processes, employees demonstrate more conservative tendencies, reflected in their adherence to current status quo and resistance to trying new methods and ideas. This tendency hinders individual growth in creative thinking domains and ultimately limits the improvement of employees' creative performance (Purc and Laguna, 2019).

Based on this reasoning, the following hypothesis is proposed:

H2a: Promotion focus is positively related to employees' creative performance.

H2b: Prevention focus is negatively related to employees' creative performance.

#### 2.2.3 The moderated mediating effect of work-specific regulatory focus

Integrating the theoretical logic of Cognitive Appraisal Theory (CAT) and Regulatory Focus Theory (RFT), this study constructs a moderated mediating model to clarify the indirect mechanism through which overwork climate impacts employees' creative performance. Specifically, work-specific regulatory focus serves as a dual mediating pathway, while trait mindfulness functions as a boundary condition that modulates the entire transmission process. The core logic of this model is as follows: trait mindfulness, as an individual difference variable (Tang and Braver, 2020), shapes employees' cognitive appraisal of the overwork climate—that is, weighing the ‘potential gains' against the ‘potential losses' embedded in the climate. This differentiated cognitive appraisal further activates distinct work-specific focus orientations (H1a and H1b), which in turn exert heterogeneous effects on employees' creative performance (H2a and H2b). Thus, the indirect influence of overwork climate on employees' creative performance via work-specific regulatory focus is contingent upon the level of trait mindfulness, forming a complete moderated mediating mechanism.

When employees exhibit high trait mindfulness, their enhanced cognitive regulation capacity and non-judgmental present-moment awareness enable them to objectively perceive the overwork climate. Rather than being overwhelmed by the climate and generating instinctive stress responses, they are able to acutely identify the potential gains within this overall climate. Specifically, the extra effort required in an overwork climate is often closely associated with potential gains such as promotions, salary increases, skill enhancement, and the realization of career goals (Montani et al., 2020). This appraisal of potential gains effectively activates a promotion focus, a motivational orientation centered on pursuing gains, growth, and aspirational goals (Pei et al., 2024). Guided by promotion focus, employees are more willing to engage in risk-taking, exploratory behaviors, and iterative trial-and-error, which are inherently congruent with the core requirements of innovation (Tao et al., 2023). As posited in H2a, promotion focus positively predicts employees' creative performance. Therefore, for employees with high trait mindfulness, the mediating role of promotion focus in the relationship between overwork climate and creative performance is more salient. Based on this reasoning, the following hypothesis is proposed:

H3a: Promotion focus mediates the positive relationship between overwork climate and employees' creative performance, and this mediating effect is moderated by trait mindfulness—specifically, the mediating effect is stronger when employees' trait mindfulness is high.

In contrast, employees with low trait mindfulness lack sufficient cognitive resources to effectively cope with stressors and are prone to automatic, reactive cognitive patterns (Molina et al., 2025). This prevents them from rationally evaluating the overall characteristics of the overwork climate. Consequently, they are more likely to magnify the potential losses embedded in the overwork climate, such as physical and mental health issues, work-family conflict, and job insecurity. This appraisal of potential losses strongly activates a prevention focus—a motivational orientation focused on avoiding losses, fulfilling obligations, and maintaining safety (Pei et al., 2024). As noted in RFT, employees in a prevention focus state exhibit conservative, risk-averse behaviors, such as adhering rigidly to established routines, avoiding untested methods, and suppressing creative ideas that may involve uncertainty (Lanaj et al., 2012). These behavioral tendencies directly conflict with the exploratory and norm-breaking nature of innovation, thereby inhibiting employees' creative performance. Consequently, for employees with low trait mindfulness, the mediating role of prevention focus in the relationship between overwork climate and creative performance is more prominent. Based on this reasoning, the following hypothesis is proposed:

H3b: Prevention focus mediates the negative relationship between overwork climate and employees' creative performance, and this mediating effect is moderated by trait mindfulness—specifically, the mediating effect is stronger when employees' trait mindfulness is low.

**Figure 1:**
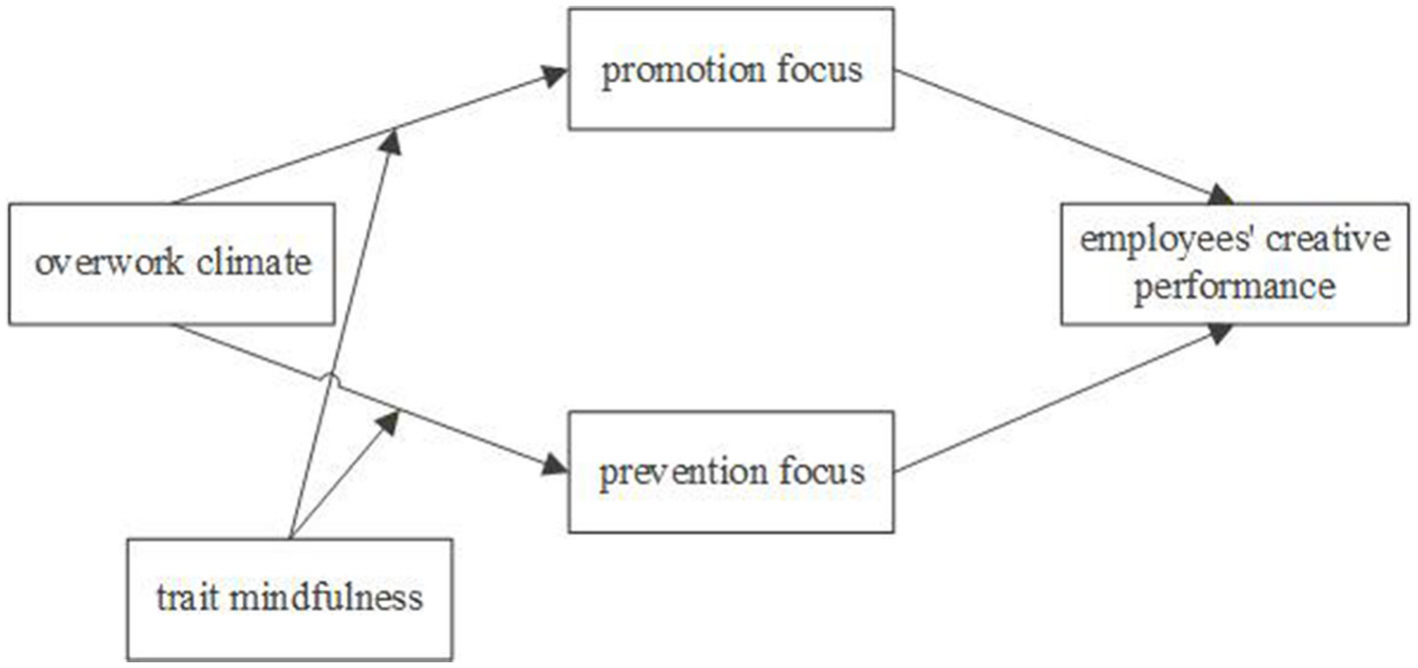
Theoretical model diagram.

The theoretical model diagram (Figure 1) of this study is as follows.

## 3 Methodology

### 3.1 Sample and procedure

To test the proposed hypotheses, a two-stage questionnaire survey was conducted targeting R&D staff in Chinese technology firms, as their roles are central to creative performance and are often subject to intense overwork climate due to the competitive nature of the industry (Chen et al., 2023). R&D staff were selected because their work inherently requires creative problem-solving and innovation, making them an ideal population to study the impact of overwork climate on creative performance.

The survey was administered through the Credmo platform, a widely used online survey tool in China, known for its ability to reach diverse professional populations and ensure high-quality responses through verified participant pools. Following the platform's protocol, questionnaires were randomly distributed to eligible participants based on the study's criteria. Prior to large-scale data collection, a pilot study was conducted with a small sample to assess the questionnaire's quality. Feedback from this pre-survey informed adjustments to item wording and order, thereby enhancing the instrument's validity.

The formal survey was conducted in two waves. The first wave, initiated in October 2023, involved distributing 730 questionnaires, yielding 679 responses (response rate: 93.01%). This wave captured data on the independent variable (overwork climate), the moderating variable (trait mindfulness), and demographic information such as employees' gender, age, educational background and organizational tenure. The second wave was conducted 3 months later. Regarding the time interval between the independent and dependent variables, there is no uniformity in the existing studies. In this study, the time interval was set at 3 months based on the purpose of establishing causal ordering, minimizing common method bias, and balancing trait stability with memory effects in social science longitudinal designs (Ployhart R and Vandenberg R, 2010). This approach facilitates observation of the impact of overwork climate on work-specific regulatory focus and creative performance. Using the platform's data cleaning function, we import the subjects from the previous survey into the sample pool, and in order to enable a secondary follow-up of the same participants, we pushed the questionnaire again to a specific list of participants in the sample pool. At this stage, the questionnaire was re-administered to the initial 679 respondents to collect data on the mediator (promotion focus and prevention focus) and the dependent variable (employees' creative performance), resulting in 621 returned surveys. Following data collection, responses from both waves were matched. Invalid questionnaires with missing data or inconsistent answers were eliminated, yielding a final sample of 597 valid, matched responses for analysis.

The descriptive statistics of the samples are as follows. The sample consisted of 56.3% males and 43.7% females. The age distribution was as follows: 16.8% were under 25, 72.7% were between 26 and 35, 8.2% were between 36 and 45, and 2.3% were over 46. From the perspective of the education level, there are 57 respondents with college degree or below (9.5%), 463 respondents with bachelor degree (77.6%), 70 respondents with master degree (11.7%) and 7 respondents with doctor's degree (1.2%). Overall, the sample predominantly comprises post-90s employees holding a bachelor degree or higher, characterized by strong innovation demands and capabilities, thereby meeting the study's selection criteria.

### 3.2 Measures

To ensure measurement reliability and validity, all key variables were assessed using established scales. Responses were captured on a five-point Likert scale (1= strongly disagree to 5 strongly agree).

**Overwork climate** was measured using the 7-item scale developed by Mazzetti et al. (2016), a sample item is, “Management encourages overtime work” (Cronbach's α = 0.886 in this study).

**Employees**' **creative performance** was measured using the 3-item scale from the research by Dul et al. (2011), a sample item is, “In my work, I often generate novel ideas”(Cronbach's α = 0.842 in this study).

**Work-specific regulatory focus** adopts the scale developed by Wallace and Chen (2006), consisting of 12 items, including 6 items each for promotion focus and prevention focus. Representative items for promotion focus include “getting my work done no matter what,” (6 items, Cronbach's α = 0.752 in this study); representative items for prevention focus include “following the rules and regulations” (6 items, Cronbach's α = 0.833 in this study).

**Trait mindfulness** adopts the Mindful Attention Awareness Scale developed by Brown and Ryan Brown and Ryan (2003). This scale is a unidimensional scale consisting of 15 items, such as “I may experience certain emotions without realizing it until later” (Cronbach's α = 0.940 in this study).

**Control variables**. The empirical analyses accounted for the effects of four individual features: gender (0 = female, 1 = male), age (coded into four categories: 1 = under 25, 2 = 26-35, 3 = 36–45, 4 = over 46), educational background (1 = college degree or below; 2 = bachelor's degree; 3 = master's degree; 4 = doctoral degree or above), and organizational tenure (counting by month) in company. According to the creativity component theory, domain knowledge is an essential element for generating creative performance (Amabile, 1983). Therefore, it can be inferred that education level, organizational tenure, and age are significant predictors of employees' creative performance, a finding corroborated by relevant research. Previous studies have found that, educational attainment is positively correlated with an individual's creative performance (Ng and Feldman, 2009). Employees who have accumulated experience within their current positions may exhibit a higher level of confidence in their ability to successfully generate novel ideas (De Clercq and Mustafa, 2024). Younger employees are more active in breakthrough innovation, while older employees tend to drive incremental innovation through their deep domain knowledge (Simonton D, 2007). Furthermore, the meta-analysis results indicate that, men generally perform better in creative performance of workplace (Hora et al., 2021).

## 4 Data analysis and empirical testing

### 4.1 Common method biases

Given that the data were collected via self-report measures, potential common method bias (CMB) was a concern. To mitigate this issue, procedural and statistical remedies were implemented: (1) participant anonymity was assured to reduce evaluation apprehension; (2) data were collected in two waves to increase data diversity and minimize bias associated with single-time-point measurement; (3) Harman's single-factor test was conducted to assess CMB severity. Principal component analysis of all scale items (unrotated exploratory factor analysis) revealed that the total variance explained by factors with eigenvalues greater than 1 was 56.882%. The variance explained by the first principal component was 24.816%, which is less than the 40% threshold for significant CMB. These results indicate that CMB does not substantially affect the validity of the study's findings.

### 4.2 Confirmatory factor analysis

To assess the discriminant validity of the core constructs, confirmatory factor analysis (CFA) was performed using Mplus 8.3. To optimize the ratio of sample size to estimated parameters (and given that CFA aims to distinguish between variables rather than explore item-level relationships), the measurement items of all variables except employees' creative performance were parceled. As presented in Table 1, the hypothesized five-factor model (overwork climate, promotion focus, prevention focus, trait mindfulness, employees' creative performance) exhibited the best fit to the data (χ^2^=164.924, df =80, χ^2^/df=2.062, RMSEA=0.042, SRMR=0.041, CFI=0.98, TLI=0.973). This model outperformed all alternative models (four-factor, three-factor, two-factor, and one-factor models), providing strong evidence for the discriminant validity of the core constructs. The results also support treating promotion focus and prevention focus as independent dimensions of work-specific regulatory focus in subsequent analyses.

**Table 1:** Results of confirmatory factor analysis.

Model	χ^2^	df	χ^2^/df	RMSEA	SRMR	CFI	TLI
Hypothesized five-factor model (Oc, Pro, Pre, Tm, Cp)	164.924	80	2.062	0.042	0.041	0.98	0.973
Four-factor model (Oc, Pro+Pre, Tm, Cp)	642.318	84	7.647	0.106	0.125	0.834	0.867
Three-factor model (Oc, Pro + Pre + Tm, Cp)	1,417.625	87	16.295	0.16	0.123	0.683	0.617
Two-factor model (Oc, Pro + Pre + Tm + Cp)	2,302.183	89	25.867	0.204	0.153	0.472	0.378
One-factor model (all constructs combined)	2,537.489	90	28.194	0.213	0.158	0.417	0.319

Oc, Overwork climate; Pro, Promotion focus; Pre, Prevention focus; Cp, Creative performance; Tm, Trait mindfulness; *N* = 597.

### 4.3 Descriptive statistics and correlation analysis

Table 2 presents the means, standard deviations, and correlations for all study variables. Overwork climate was significantly negatively correlated with employees' creative performance (*r* = −0.125, *p* < 0.05) and significantly positively correlated with prevention focus (*r* = 0.26, *p* < 0.001), while no significant correlation was observed between overwork climate and promotion focus. Promotion focus was significantly positively correlated with employees' creative performance (*r* = 0.409, *p* < 0.001), and prevention focus was significantly negatively correlated with employees' creative performance (*r* = −0.22, *p* < 0.001).

**Table 2:** Descriptive statistics and bivariate correlations of the study variables.

Variable	M	S.D.	1	2	3	4	5	6	7	8	9
1.Gender	0.44	0.50	1								
2. Age	1.96	0.59	0.116^*^	1							
3.Edu	2.05	0.51	0.008	0.028	1						
4. Ot	45.98	40.14	0.051	0.443^**^	−0.007	1					
5. Oc	2.71	0.94	−0.076	−0.038	0.027	−0.071	1				
6. Pro	4.00	0.54	−0.066	0.121^*^	0.023	0.118^*^	−0.064	1			
7. Pre	3.03	0.90	−0.065	−0.056	−0.023	−0.035	0.26^**^	−0.006	1		
8. Cp	4.14	0.64	0.031	0.145^**^	0.057	0.143^**^	−0.125^*^	0.409^**^	−0.22^**^	1	
9. Tm	2.16	0.74	−0.081^*^	−0.21^**^	−0.072	−0.125^*^	0.355^**^	−0.374^**^	0.344^**^	−0.47	1

^*^p < 0.05, ^**^p < 0.01, Gender (0 = male, 1 = female); Age (1 = under 25, 2 = 26-35, 3 = 36-45, 4 = over 46); Edu(indicating education level, 1 = college degree or below, 2 = bachelor's degree, 3 = master's degree, 4 = doctoral degree or above); Ot indicates the tenure of the organization; *N* = 597.

### 4.4 Hypotheses testing

Hierarchical regression analysis and bootstrap analysis conducted with SPSS were used to test the hypotheses, with results presented in Tables 3, 4.

**Table 3:** Regressive analysis.

Variable	Promotion focus		Prevention focus		Employees' creative performance				
	Model 1	Model 2	Model3	Model 4	Model 5	Model 6	Model 7	Model 8	Model 9
Gender	−0.087^*^	−0.099^**^	−0.040	−0.032	0.003	0.044	0.037	−0.001	−0.005
Age	0.095^*^	0.022	−0.042	0.012	0.098^*^	0.061	0.061	0.089^*^	0.090^*^
Edu	0.024	−0.012	−0.029	0.002	0.058	0.046	0.049	0.051	0.053
Ot	0.077	0.064	0.003	0.017	0.092^*^	0.068	0.062	0.097^*^	0.093^*^
Oc	−0.062	0.104^*^	0.257^***^	0.115^**^	−0.117^**^		−0.093^*^		−0.067
Pro						0.394^***^	0.388^***^		
Pre								−0.210^***^	−0.192^***^
Tm		−0.432^***^		0.343^***^					
Oc × Tm		0.096^*^		−0.138^**^					
Adjusted R^2^	0.023	0.158	0.064	0.147	0.037	0.183	0.183	0.068	0.070
F	3.765^***^	16.897^***^	9.203^***^	15.676^***^	5.623^***^	26.441^***^	23.252^***^	9.655^***^	8.515^***^

^*^p < 0.05, ^**^p < 0.01, ^***^p < 0.001; *N* = 597.

**Table 4:** Bootstrap-based test of moderated mediating effects.

Pathway	Effect size	Standard error	95% Bootstrap CI (lower bound)	95% Bootstrap CI (upper Bound)
Overwork Climate → Promotion Focus				
– Low trait mindfulness	0.0079	0.0279	−0.0469	0.0627
– High trait mindfulness	0.1334	0.0425	0.0500	0.2167
Overwork climate → prevention focus				
– Low trait mindfulness	0.2366	0.0463	0.1456	0.3276
– High trait mindfulness	−0.0449	0.0705	−0.1835	0.0936
Overwork climate → promotion focus → employees' creative performance				
– Low trait mindfulness	0.0038	0.0135	−0.0258	0.0278
- High trait mindfulness	0.0637	0.0242	0.0198	0.1146
Overwork climate → prevention focus → employees' creative performance				
- Low trait mindfulness	−0.0346	0.0097	−0.0559	−0.0179
- High trait mindfulness	−0.066	0.0097	−0.0126	0.0263

**Figure 2:**
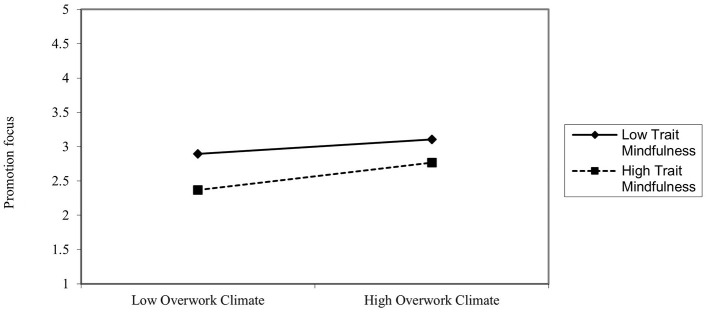
The moderating effect of trait mindfulness on overwork climate and promotion focus.

Hypothesis H1a predicted that the interaction between overwork climate and trait mindfulness would positively influence promotion focus, with a stronger effect for employees with higher trait mindfulness. As shown in **Model 2** of Table 3, the interaction term (overwork climate × trait mindfulness) had a significant positive effect on promotion focus (β = 0.096, *p* < 0.05). The results in Table 4 show bootstrap analysis results: when trait mindfulness was at a low level, the interaction term had no significant effect on promotion focus [β = 0.0079, 95% CI = (−0.0469, 0.0627)]; when trait mindfulness was at a high level, the interaction term had a significant positive effect on promotion focus [β = 0.1334, 95% CI = (0.0500, 0.2167)]. Additionally, the simple effects plot (Figure 2) demonstrates that higher trait mindfulness levels amplify the influence of overwork climate on promotion focus. These results support Hypothesis H1a.

**Figure 3:**
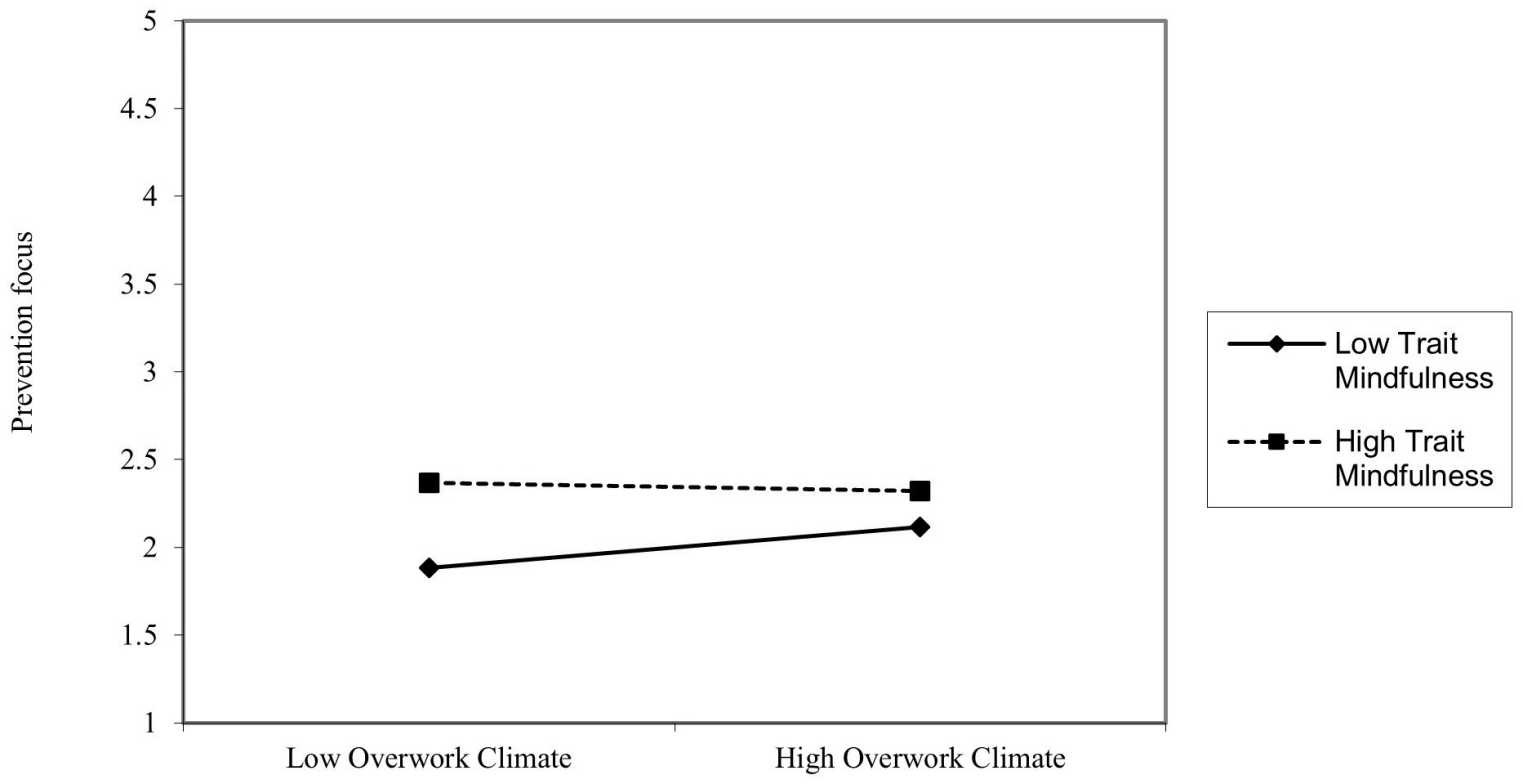
The moderating effect of trait mindfulness on overwork climate and prevention focus.

Hypothesis H1b predicted that the interaction between overwork climate and trait mindfulness would negatively influence prevention focus, with a stronger effect for employees with lower trait mindfulness. As shown in **Model 4** of Table 3, the interaction term (overwork climate × trait mindfulness) had a significant negative effect on prevention focus (β = −0.138, *p* < 0.01).The results in Table 4 show, when trait mindfulness was at a high level, the interaction term had no significant effect on prevention focus [β = −0.0449, 95% CI= (−0.1835, 0.0936)]; when trait mindfulness was at a low level, the interaction term had a significant positive effect on prevention focus [β = 0.2366, 95% CI = (0.1456, 0.3276)]. Figure 3 further illustrates that the positive influence of overwork climate on prevention focus intensifies with decreasing trait mindfulness levels. These findings validate Hypothesis H1b.

Hypothesis H2a predicted a positive correlation between promotion focus and employees' creative performance. As shown in **Model 6** of Table 3, promotion focus had a significant positive effect on employees' creative performance (β = 0.394, *p* < 0.001), supporting Hypothesis H2a.

Hypothesis H2b predicted a negative correlation between prevention focus and employees' creative performance. As shown in **Model 8** of Table 3, prevention focus had a significant negative effect on employees' creative performance (β = −0.210, *p* < 0.001), supporting Hypothesis H2b.

Hypotheses H3a and H3b predicted that promotion focus and prevention focus would mediate the interaction effect of overwork climate and trait mindfulness on employees' creative performance, with the mediating effect varying by trait mindfulness level. Following Edwards and Lambert's methodology, bootstrap analysis was used to calculate 95% confidence intervals for the indirect effects at ± 1 standard deviation of trait mindfulness.

For Hypothesis H3a (mediating role of promotion focus), we can see this result in Table 4. When trait mindfulness was at a high level, the indirect effect of overwork climate on employees' creative performance via promotion focus was significant [β = 0.0637, 95% CI = (0.0198, 0.1146)]; when trait mindfulness was at a low level, the indirect effect was not significant [β = 0.0038, 95% CI = (−0.0258, 0.0278)]. The difference between the two indirect effects was significant [β = 0.0637, 95% CI = (0.0068, 0.1271)], indicating that the mediating effect of promotion focus was stronger for employees with higher trait mindfulness. These results support Hypothesis H3a.

For Hypothesis H3b (mediating role of prevention focus), When trait mindfulness was at a low level, the indirect effect of overwork climate on employees' creative performance via prevention focus was significant [β = −0.0346, 95% CI = (−0.0559, −0.0179)]; when trait mindfulness was at a high level, the indirect effect was not significant [β = −0.0066, 95% CI = (−0.0126, 0.0263)]. The difference between the two indirect effects was significant [β = 0.0411, 95% CI = (0.0166, 0.0713)], indicating that the mediating effect of prevention focus was stronger for employees with lower trait mindfulness. These results support Hypothesis H3b.

## 5 Conclusion and discussion

### 5.1 Research results

In the current era of “involution,” with the mobility and digitization of the workplace and intensified competition among enterprises, many enterprises and employees are improving performance and productivity through overwork, leading to a strong overwork climate in enterprises and posing many hidden dangers to the physical and mental health of employees (Kobayashi et al., 2024). In order to better balance the development needs of enterprises and the physical and mental health of employees, this study focuses on R&D personnel in Chinese technology companies and uses a questionnaire survey to examine the impact of overwork climate on their creative performance. The findings are as follows:

First, trait mindfulness serves as a crucial boundary condition influencing the effect of overwork climate. The interaction between overwork climate and trait mindfulness affects employees' situational regulation focus. When employees have higher trait mindfulness levels, overwork climate may activate their promotion focus; conversely, they may trigger prevention focus. This finding suggests that the influence of overwork climate on work-specific regulatory focus is contingent upon their trait mindfulness levels.

Second, employees' promotion focus is positively correlated with creative performance, while prevention focus is negatively correlated. Specifically, when employees' behavioral motivation leans toward seeking benefits, they tend to engage in challenging tasks, thereby demonstrating higher creative performance. Conversely, when their motivation leans toward avoiding loss, they are more likely to adhere to rules, resulting in relatively lower creative performance.

Third, the interaction between overwork climate and trait mindfulness levels influences employees' creative performance through the mediating role of regulation focus. Higher trait mindfulness levels strengthen the mediating effect of promotion focus, while lower trait mindfulness levels amplify the mediating effect of prevention focus. This indicates that the mediating mechanism of overwork climate on creative performance varies across different trait mindfulness levels. When employees exhibit higher trait mindfulness levels, the overwork climate activates their promotion focus, thereby enhancing creative performance. Conversely, when employees have lower trait mindfulness levels, the overwork climate activates prevention focus, thereby reducing creative performance. This aligns with the findings of scholars such as Wallace and Chen (2006), who argue that work-specific regulatory focus serves as a proximal motivational mediator between distal antecedents (e.g., personality traits, organizational climate) and performance outcomes, elucidating how individual and situational factors influence job performance.

It is worth noting that the empirical results indicate that an overwork climate is generally negatively correlated with employees' creative performance (See Table 3 for **Model 5**). We argue that this finding is not inconsistent with the existence of a positive mediating mechanism for the overwork climate. From a statistical perspective, the directions of direct and indirect effects are not necessarily consistent, they reflect different pathways and mechanisms through which the independent variable influences the dependent variable (Bellavia and Valeri, 2018). In this study, as an independent variable, the overwork climate exhibits a direct negative effect on employees' creative performance. In addition to the prevention focus confirmed in this study, this effect may also stem from fatigue, resource depletion, and interpersonal relationship problem (Mazzetti et al., 2016) resulting from excessive work under an overwork climate. However, at the same time, an overwork climate may also produce a positive indirect effect by activating employees' promotion focus as a mediating variable. This is because, under specific conditions (such as when employees have a high trait mindfulness), they may perceive the potential benefits that an overwork climate might bring to the organization, such as access to more talented peers, opportunities for professional growth, and promotions opportunity (Mazzetti et al., 2016). The study by Zeng further supports this perspective opportunity (Zeng et al., 2025). They found that an overwork climate has a more significant positive impact on the innovation performance of researchers with lower professional titles.

In addition to individual properties, cultural context may also act as a crucial moderating variable for the impact of the overwork climate. For instance, research has found that in collectivist cultures, employees exhibit a higher tolerance for overwork climate (Takagi et al., 2025). However, in individualist cultures, employees tend to protect themselves by reducing their effort when faced with overwork climate (Wojtczuk-Turek et al., 2024). Consequently, in regions where collectivism is more prevalent, the promotional effect of an overwork climate on employees' creative performance may be amplified. Furthermore, power distance may also serve as a key boundary condition influencing the “overwork climate—regulatory focus—employees' creative performance” relationship. Employees in cultures with higher power distance are more likely to view leaders' requests for overtime as reasonable (Oruh and Dibia, 2020), thereby proactively adjusting their work state to engage in innovative activities, which in turn reinforces the promotional effect of overwork climate on employees' creative performance.

### 5.2 Theoretical value

First, this study integrates Cognitive Appraisal Theory with the Regulatory Focus Theory, effectively resolving the issue of ambiguous stress evaluation criteria in Cognitive Appraisal Theory. In existing research on stress perception, scholars have primarily focused on dimensions such as the nature of the street themes (Zhang et al., 2019) and the availability of stress coping resources. However, the current mainstream Challenge–Hindrance Stressor Framework exhibits a certain degree of mechanistic rigidity, its binary classification of stress is overly absolute, making it difficult to account for the heterogeneity of individual stress perceptions (Podsakoff N et al., 2023). In the meantime, stress perception evaluation from a resource perspective suffers from limitations such as the fragmentation of resource dimensions and the individual heterogeneity in how resource gains and losses influence the assessment of stressors (Cunningham, 2025). To address these theoretical shortcomings, this study systematically integrates the “gain-loss” analysis framework from the Regulation Focus Theory into the process of employees' perception and evaluation of overwork climates. By employing the core logic of gain-oriented and loss-oriented perspectives, it defines the cognitive evaluation criteria for overwork climate, thereby fundamentally addressing the long-standing unresolved core question in Cognitive Appraisal Theory: “What are the evaluation criteria?” This integration not only provides Cognitive Appraisal Theory with a new analytical perspective and a unified evaluative logic, effectively addressing the theory's limitations in specific stress scenarios, but also promotes cross-theoretical integration between Cognitive Appraisal Theory and Regulation Focus Theory.

Second, this study identifies trait mindfulness as a critical boundary condition for the stress appraisal of perceived overwork climate, thereby enriching the contingent perspective in overwork and employees' creative performance research. Although existing literature has explored the interactive effects of overwork climate with individual traits such as perfectionism (Mazzetti et al., 2014), it has rarely identified which dispositional factor determines employees' appraisal and response of overwork climate stimuli. This study verifies trait mindfulness as a key moderator that shapes how employees interpret and evaluate overwork, which effectively explains heterogeneous creative performance under identical overwork contexts and supplements the missing boundary condition in prior research.

Third, this study unpack the dual effects of perceived overwork climate on creative performance, providing a more systematic and inclusive explanatory framework. Prior studies have yielded inconsistent conclusions by focusing on the unilateral effect of overwork climate (Song et al., 2026; Zeng et al., 2025; Mazzetti et al., 2016). The proposed model integrates situational stimuli, individual traits, stressors self-regulatory strategies, and creative outcomes, systematically explaining why overwork climate exerts both facilitating and inhibiting influences on employees' creative performance. This framework overcomes the one-sidedness of earlier research and strengthens the explanatory power of “overwork climate–creative performance” research.

Finally, this study identifies the positive effects of an overwork climate in specific contexts, thereby enriching the body of contextual research on this topic. Previous studies on overwork climate have largely been based on Western samples (Lazauskaite-Zabielske et al., 2023; Žiedelis et al., 2024), and while a few have focused on the Chinese context (Song et al., 2026; Zeng et al., 2025), there is a lack of research specifically targeting R&D personnel in technology-based companies with overwork climate. This study used this specific group as its sample and found that a overwork climate not only activates employees' prevention focus but may also stimulate their promotion focus under specific circumstances. This study suggests that, in addition to individual factors (such as trait mindfulness), this may be due to the fact that China is generally a country that emphasizes collectivism and has a high power distance (Ma et al., 2016). In this context, employees are more likely to accept strong management and high work demands and do not view them as factors that undermine psychological safety (Yin et al., 2022). Consequently, employees are more receptive to overwork climate and proactively adjust their work states to engage in innovative activities. This finding expands the existing body of research on overwork climate within Eastern cultural contexts and further enriches the contextualized research on this topic.

### 5.3 Practical insights

Based on the above research conclusions, we provide the following management recommendations.

**Firstly, revisiting perceptions of overwork culture and rejecting the “overtime-only” mentality**. This study reveals that overwork climates generally harm employees' creative performance. To address this, we must shift the perception of overwork and dispel misconceptions like “overtime equals dedication” and “long hours equal efficiency”. For a start, in internal communications, performance evaluations, and promotion incentives, the focus should transition from work hours to work quality, innovative outcomes, and value contribution. This approach reinforces the principle that “effective effort outweighs futile accumulation”, reducing meaningless overtime and superficial competition. Additionally, scientifically set work objectives and timelines based on job characteristics and task complexity to avoid blind pressure. Establish reasonable work hour limits (e.g., strictly controlling weekly overtime to statutory standards) and prohibit unpaid overtime and forced overtime. Finally, implement compensation mechanisms like compensatory leave or salary adjustments for urgent project-related overtime to reduce employees' resistance.

**Secondly, activating the promoting focus and cultivating the soil for innovation**. Research reveals that fostering a promotion focus significantly enhances creative performance, while a prevention focus has a negative impact. To cultivate this mindset, companies should implement institutional designs and cultural initiatives to guide employees toward a breakthrough-driven attitude. To begin with, establish an innovation rewards and failure tolerance mechanism. By creating dedicated innovation awards, companies can recognize employees who propose novel solutions or drive technological improvements through both material incentives and recognition, reinforcing a reward-oriented motivation. Furthermore, cultivate an error-tolerant management culture by clearly defining acceptable failures. This prevents excessive blame for minor mistakes, reduces employees' fear of failure, and encourages risk-taking. In addition, strengthen growth-oriented goal-setting, incorporate innovation targets into performance metrics, linking them to career advancement and training opportunities. This shifts employees' focus from merely error avoidance to self-development and achievement fulfillment. Last but not least, provide innovation resources and platforms. Establish innovation labs and cross-departmental teams to create spaces for idea exchange and practical experimentation. Ensure sustained investment in innovation resources, encouraging employees to acquire new knowledge and adopt novel approaches. This enhances their innovation capabilities and confidence, further solidifying the promotion focus.

**Finally, enhancing employees' mindfulness levels and stress adaptability**. Mindfulness can be developed through structured training programs. Organizations can implement systematic measures to help employees improve cognitive patterns and strengthen their ability to positively cope with excessive work pressure: First and foremost, conduct mindfulness training and psychological support programs, regularly organizing mindfulness courses and stress management workshops. Teach employees to handle work pressure through “non-judgmental, present-focused” approaches to reduce self-sacrifice; Moreover, foster a corporate culture emphasizing physical and mental health, encouraging employees to take breaks during work and advocating for work-life balance to avoid the value of sacrificing health for performance; Besides, promote mindfulness concepts through internal communication to help employees recognize the importance of accepting pressure and responding efficiently; Ultimately, Leaders should exemplify mindfulness by demonstrating calm stress management in their work, avoiding passing anxiety to employees. Emphasize listening and understanding in communication, refrain from critical evaluations, and set mindfulness role models to subtly influence employees' cognition and behavior.

## 6 Research limitations and future directions

### 6.1 Research limitations

First, regarding variable measurement, although this study mitigated the issue of common variance to some extent by employing a two-wave design with a three-month lag, limitations remain in terms of measurement. These include the simultaneous measurement of overwork climate and trait mindfulness, the failure to distinguish between long-term antecedent variables and short-term mediating variables, and the use of self-report questionnaires, all of which may affect the robustness of the study's conclusions. This study measured trait mindfulness (the moderator) and perceived overwork climate (the independent variable) at the same time point (T1), posing a potential risk of bidirectional causality. Additionally, the study measured the moderating focus only once, making it impossible to determine whether it represents a momentary state or a relatively stable individual trait formed under conditions of overwork climate. All of these factors may interfere with causal inferences within the theoretical model.

Second, regarding sample selection, the study's sample primarily consists of R&D personnel within domestic Chinese technology companies. While this focus facilitates an in-depth analysis of key characteristics and mechanisms in a specific context, it also introduces certain limitations. Specifically, given the relatively concentrated geographical, industry, and cultural backgrounds of the sample, the external validity and generalizability of the findings and theoretical inferences may face challenges when attempting to generalize them to contexts in other countries, different industrial sectors, or diverse organizational structures.

Finally, this model examines trait mindfulness solely as a boundary condition at the individual level, without considering the cross-level moderating effects of cultural dimensions on the “overwork climate–regulatory focus–creative performance” pathway. Existing research indicates that the impact of overwork climate varies across different cultural contexts (Takagi et al., 2025). China is a country that emphasizes collectivism and power distance, where employees may be more accepting of an overwork environment. In cultures with lower individualism and power distance, the intensity and mechanisms by which an overwork climate affects employees' creative performance may differ.

### 6.2 Future prospects

#### 6.2.1 Future improvements could be made in the following areas

First, optimize the research design and variable measurement methods by collecting data through multi-time-point, multi-source approaches, conducting repeated measurements of variables across multiple time points, and employing methods such as daily tracking studies based on the Empirical Sampling Method (ESM) and cross-lagged models to enhance the robustness of the research findings.

Second, the sample scope should be expanded to explore the validity of this study's findings across different cultural contexts, industries, and occupational groups, thereby further validating the generalizability of the research model.

Third, we will investigate the cross-level moderating effects of cultural contexts, such as collectivism and power distance, on the “overwork climate–regulatory focus–creative performance” pathway. We will examine the boundary effects of cultural dimensions within this framework to further clarify the contextual conditions under which an overwork climate influences creative performance, thereby deriving research conclusions that are both more universally applicable and better aligned with diverse cultural contexts.

## Data Availability

The raw data supporting the conclusions of this article will be made available by the authors, without undue reservation.
